# Editorial: Advances in diagnosing and treating new-onset refractory status epilepticus (NORSE)

**DOI:** 10.3389/fneur.2023.1270702

**Published:** 2023-08-30

**Authors:** Aljoscha Thomschewski, Giada Giovannini, Nicolas Gaspard, Mirja Steinbrenner, Ronny Wickström, Julia Jacobs

**Affiliations:** ^1^Department of Neurology, Christian-Doppler University Hospital, Paracelsus Medical University, Salzburg, Austria; ^2^Unitá di Neurologia, Azienda Ospedaliera Universitaria di Modena, Modena, Italy; ^3^Hôpital Erasme, Université Libre de Bruxelles, Brussels, Belgium; ^4^Clinic for Neurology With Experimental Neurology, Charité University Medicine Berlin, Berlin, Germany; ^5^Department of Women's and Children's Health, Karolinska Institutet, Solna, Sweden; ^6^Alberta Children's Hospital, University of Calgary, Calgary, AB, Canada

**Keywords:** epilepsy, new-onset refractory status epilepticus, NORSE, refractory status epilepticus, febrile infection-related epilepsy syndrome, FIRES, neuroinflammation, pathophysiology

New-onset refractory status epilepticus (NORSE) is a rare but devastating condition describing a group of diseases and disorders that are characterized by *de novo* onset of uncontrollable seizures, so called refractory status epilepticus (RSE), without an identifiable acute or active structural, toxic, or metabolic cause (1). Febrile infection-related epilepsy syndrome (FIRES) is a subcategory of NORSE with the addition of prior febrile infection between 2 weeks and 24 h before the onset of RSE (1, 2).

Reliable numbers regarding its incidence are lacking, but occurrence has been estimated to be ~1–2 per 100,000 person years (3, 4). In case an etiology is found, such as autoimmune encephalitis, treatment can be directed at the underlying pathophysiology. However, therapeutic effects are often not sufficient, and in ~75% of patients, the underlying etiology remains unknown (5–7). Although plenty of studies have been published on NORSE and FIRES over the last decades, no high-level evidence exists on which to base diagnostic and treatment recommendations. Given the very poor prognosis with a mortality rate of 10–30% and severe sequelae in most surviving patients (8), this lack of evidence is troublesome. Hence, this Research Topic aimed at collecting novel research and findings on the matter in order to ease the effort of systematically assessing the body of evidence in future.

A detailed description of the clinical, etiological, electrophysiological neuroimaging and outcomes of new onset status epilepticus (SE) cases and their differences from SE developing in epileptic patients is reported by Benaiteau et al.. As NORSE/FIRES identifies a “clinical presentation,” it could be sustained by many different etiologies, and more than 200 uncommon disorders have been described so far (9). Inflammatory/autoimmune and paraneoplastic causes reach up to 40% of all etiologies representing the most relevant group followed by infective unusual causes that represents up to 10%, while genetic, metabolic, and toxic causes are considered rare (10). However, assessing the genetic landscape of NORSE/FIRES is an expanding field parallel to the improvement of the genetic tests and to the increase the knowledge of pathogenic variants related to epilepsy and SE in general.

Among genetic causes, mitochondrial diseases could play an important role both in children and adults. Astner-Rohracher et al. reported on a new pathogenic variant in FASTK2D presenting as NORSE in a young patient thus extending the phenotypical spectrum of FASTKD2-related mitochondrial disease. Overall, the role of genetic etiologies is evaluated by deCampo et al. in a single-center retrospective study on 25 children with FIRES over a 10-year period. None of the tests resulted as positive/causative, thus confirming the rarity of the genetic etiology. Nevertheless, the authors underline also the important aspect of the heterogenicity of the tests, thus giving a focus and a critical view on new diagnostic perspectives (deCampo et al.).

The diagnostic workup in general is a central aspect in NORSE/FIRES. In fact, it is known that after an extensive diagnostic work-up, ~50% of cases remain with unknown etiology, thus representing “cryptogenic NORSE.” Standardized and shared diagnostic algorithms are the basis for improving the management and trying to reduce the number of cryptogenic cases. In this view, recently, international consensus-based recommendations have been published by the International NORSE consensus group (11, 12). Sheikh and Hirsch reported and evaluated the recommendations for the in-hospital management of NORSE/FIRES patients in specialized centers, while an algorithm for the rapid identification and transfer of NORSE/FIRES patients to the most appropriate specialized center to ensure a rapid and appropriate treatment is discussed by Vinette et al..

Correct and rapid diagnoses are key in order to plan the appropriate treatment. As described by deCampo et al., there is now a trend toward increased use of immunomodulatory agents next to steroids and intravenous immunoglobulins as the most common treatment. It is important to note, however, that multiple agents are available and even more could be repurposed, making pre-clinical research immanent to further treatment options. To this end, the role of immunotherapy is addressed in the study of Cerovic et al., who developed an *in vitro* model of the mouse hippocampal/temporal cortex where epileptiform activity and drug-resistant seizures are exacerbated by neuroinflammation induction. In this model, the application of two immunomodulatory agents - anti-IL6 and anti-IL1—delayed the onset of epileptiform events and strongly reduced the ASM-resistant epileptiform activity. Their validated model highlights the therapeutic potential of anti-inflammatory agents in NORSE/FIRES (Cerovic et al.).

In addition to drug therapy, Sheikh and Hirsch mentioned the efficacy of ketogenic diet after early treatment with first-line immunotherapy. This notion is also supported by Nabbout et al., presenting a study of 16 patients treated with ketogenic diet. Next to their patients, the authors present a systematic meta-analysis of the published case, concluding high efficacy of ketogenic diet in patients with general RSE (with half the patients experiencing RSE cessation in 7 days) and also patients with NORSE in particular (Nabbout et al.).

In addition to medical drugs and ketogenic diet, neuromodulation offers an exciting possibility to treat NORSE as outlined by Jindal et al., Ritter and Selway, and Stavropoulos et al.. As pointed out by Ritter and Selway, vagal nerve stimulation (VNS) can be a viable treatment option, not only aiding status cessation but also enabling physicians to wean of anesthesia. Based on a systematic literature assessment as well as on two own cases, they describe several beneficial effects of VNS treatment in the chronic but notably also the acute phase of NORSE (Ritter and Selway). Of interest, one of the mentioned cases with VNS implantation from the same group is described in more detail by Jindal et al. further revealing that VNS can be safely used in a pregnant patient with NORSE. On a broader spectrum, the usage of VNS as well as electroconvulsive therapy and deep brain stimulation is investigated as part of a systematic literature review by Stavropoulos et al., showing that any of these could add to a successful treatment of NORSE and FIRES.

One of the ascribed benefits of neuromodulation techniques, such as VNS, is the lasting treatment of refractory epilepsy after cessation of status (Stavropoulos et al.). As revealed in a systematic review by Taraschenko et al., ~41% of adult and 57% of children with NORSE will experience refractory seizure occurrence after the acute phase of NORSE. Together with cognitive disabilities and psychiatric disorders arising at the chronic state, these results highlight the importance of the topic at hand (Taraschenko et al.). Data on FIRES presented by Shrestha et al. as well as Shi et al. is even more troublesome. Shrestha et al. analyzed a multi-center case series of FIRES patients, demonstrating severe neurocognitive impairment even when patients received state of the art therapy. Shi et al. showed a similar severe outcome in a retrospective single-center study of 11 adult patients with cryptogenic FIRES. Four of them died in hospital. Among long-term survivors, another patient died and even though all survivors reached functional independence, they developed drug-resistant epilepsy or remote recurrent SE mostly associated with permanent damage of hippocampus and needing anti-seizure medications polytherapy.

With these last contributions stressing the aforementioned severity of NORSE/FIRES, this Research Topic aimed at collecting evidence to guide treatment and future research in this field. The major importance of early recognition of the syndrome itself and the identification of possible etiologies becomes apparent throughout all of the contributions. While genetic testing might yield important information regarding causes for NORSE, common multidisciplinary approaches toward a clear diagnosis at specialized centers is of immanent importance in order to enable rapid and accurate treatment initiation, that ultimately could prevent fatal or severe outcomes.

## Author contributions

AT: Writing—original draft, Writing—review and editing. GG: Writing—original draft, Writing—review and editing. NG: Writing—review and editing. MS: Writing—review and editing. RW: Supervision, Writing—review and editing. JJ: Writing—review and editing.
